# Supplementary material for: Implementing a digital solution for patients with migraine—Developing a methodology for comparing digitally delivered treatment with conventional treatment: A study protocol
Source: PLOS Digit Health. 2024 Feb 29;3(2):e0000295. doi: 10.1371/journal.pdig.0000295 (PMC10903846; doi:10.1371/journal.pdig.0000295)
Supplement: S1 Text — (DOCX) [file pdig.0000295.s001.docx]

**List of abbreviations**

NI – non-pharmacological interventions

TBI – technology-based interventions

CBT – cognitive-behavioral therapy

DTx – digital therapeutics

DHD – digital headache diary

PD – paper headache diary

HIT-6 – Headache Impact Test

ESQ-2 – Emotional State Questionnaire

DSM-V – Diagnostic and Statistical Manual of Mental Disorders, Fifth Edition

SSP – Swedish Universities Scales of Personality

CPAQ-R – Chronic Pain Acceptance Questionnaire Revised

PASS – Pain Anxiety Symptoms Scale

HALT – Headache Attributed Lost Time

HURT – Headache Under-Response to Treatment
