# Supplementary material for: Implementing a digital solution for patients with migraine—Developing a methodology for comparing digitally delivered treatment with conventional treatment: A study protocol
Source: PLOS Digit Health. 2024 Feb 29;3(2):e0000295. doi: 10.1371/journal.pdig.0000295 (PMC10903846; doi:10.1371/journal.pdig.0000295)
Supplement: Attachment — Submitted filename: Response to Reviewers.pdf [file pdig.0000295.s002.pdf]

Dear Reviewers

We are sincerely grateful for all the reviewers for their time, comments and expertise on the manuscript "Implementing a digital solution for patients with migraine - developing a methodology for comparing digitally delivered treatment to conventional treatment: A study protocol" by Triinu Niiberg-Pikksööt et al.

Reviewer #1 points out generalizations made in the article that really need clarification.
Examples:
Lines 50-52: "...patients access to treatment options is limited because there are few specialists worldwide". Is that truth? How many neurologists, general physicians, pain specialists, anesthesiologists dedicated to treat pain, nurses that assisting in pain management are worldwide?

To be more precise, we correct the wording and specify that we mean specialists specializing in headaches.
Corrected sentence: Lines 50-52: "As far as we know, there is a shortage of headache specialists considering the treatment needs"

Lines 57-58: "...high quality digitally mediated interventions are the future of chronic disease treatment", is that truth in all chronic diseases?

This sentence reflects the opinion of researchers in the rapidly digitizing world and healthcare sector. However, we understand the overgeneralization of the sentence and to be more precise, we correct the wording and specify the sentence as follows: Lines 57-58: "We believe that remote monitoring and high quality digitally mediated interventions may be the future of many chronic disease treatment".

Lines 62-53: ".... Migraine is one of the most expensive diseases in the world". How many are the most expensive diseases in the world and what number does migraine occupy?

The entire paragraph with references reflects the costliness of migraine as a disease. We have brought Europe as an example, because the studied population is the closest to Europe. There are plenty of articles in the databases about the cost of migraine in different countries, and we made a choice not to refer to all the different articles here. We express our sincere gratitude for Your keen observation and bringing this matter to our awareness. We corrected the text accordingly: "Migraine is an expensive disease, annually costing the European economy 95 billion Euros".

In lines 135 – 140, the authors explain that it is difficult to get patient adherence to CBT it is delivered digitally because there is no contact or it is minimal, or because there is anxiety and depression. In the protocol the mention that one of the exclusion criteria is depression, but why they support digitally delivered CBT if they are saying in lines 135-140 that one of the no-adherence reasons is that there is no contact or it contact is minimal.

Depression is highly comorbid with migraine, so we cannot completely remove depressed patients from the study population. The exclusion criterion is indeed depression to a degree that may prevent the patient from participating in the intervention. Therefore, we have also involved a psychiatrist in the inclusion of patients, who, in addition to a neurologist, evaluates the patient's suitability for the study.
Previous studies have shown that the average user may abandon digital CBT after the first session due to the difficulty of maintaining treatment adherence with minimal or without any clinical contact. Taking the above into account, one of the sub-goals of our study is to specify the profile of such patients. We are aware that not all patients may be suitable for a digital intervention, but to our knowledge, evidence-based data on the exact patient profile have not been previously published.

We appreciate Your bringing to our attention important flaws. We will review the entire article with the goal of reducing the number of broad statements. In an article analyzing the study's findings, we also intend to provide a comprehensive explanation of the statistical analysis portion.

We are thankful for **reviewer #2** to the feedback. The summary needs clarification on our part.
Reviewer #2: I have reviewed the protocol entitled "Implementing a digital solution for patients with migraine - developing a methodology for comparing digitally delivered treatment to conventional treatment: A study protocol". The Authors designed a study to address the clinical effectiveness of digital interdisciplinary intervention using a Migraine Buddy© application.

In the current study we use the Migrevention© application for interdisciplinary digital intervention, not the Migraine Buddy© application.

Statistical analysis should include statistical methods for causal effect inference (e.g. targeted maximum likelihood extimation). This is important to evaluate the additive effect of each arm of the study.

We acknowledge that the incorporation of statistical methods for inferring causal effects is imperative in the realm of statistical analysis. The consideration of this aspect will be duly incorporated during the analysis of the data.

We acknowledge and value the recommendation put forth by **reviewer #3** to enhance the clarity of the manuscript. We would like to express our gratitude for Your insightful and concise major and minor comments. The responses to the aforementioned comments are provided in the subsequent sections.

Major comments:
1. Please proofread the whole article again, as there are various grammatical errors or typos throughout.

Thank you for bringing this to our attention. We will conduct an additional grammatical analysis and proofreading of the manuscript.

2. It was unclear from the introduction that the study will have four conditions (each with increasing numbers of intervention components) and four control groups - it sounded like there would be one comprehensive/multi-component intervention and one control group.

Thank you for bringing this to our attention. We critically examined the introductory part of the manuscript and endeavor to provide further elaboration. Section 2.8 of the manuscript provides a comprehensive description of the study setting and randomization process, offering more intricate details.

*3. The study's results will contribute to an important gap in the literature (few studies on *comprehensive* digital interventions for migraine, as well as few studies on the "dose" of each intervention component necessary), but this is not explicitly stated or highlighted as much as it should, leaving the reader confused as to what this study would specifically contribute to the literature. The authors should state/highlight this point more in both the introduction and the discussion sections.*

We sincerely appreciate your recognition of the crucial necessity to underscore the significance of the article. The study protocol has been approached with modesty. The aforementioned suggestions can be duly considered in the article that outlines the findings. Appropriate changes are made to the manuscript.

4. If there are four experimental conditions and four control groups, does that mean there will be four pair-wise comparisons for each outcome at each time point? I would suggest adding how you will adjust for multiple comparisons.

We appreciate your questions and suggestions. Each outcome is scheduled to undergo pairwise comparisons at a specific time point. We also intend to examine groups of variables using a variety of statistical approaches. If the data analysis has already been performed, subsequent articles provide a more in-depth description of methods and variables.

Minor comments:

1. Make the language clearer that the trial has not been run yet (i.e., use more future tense rather than present tense) throughout the manuscript; as the reader, it was hard to discern whether you were talking about events that had already happened, were happening presently, or will happen in the future.

We appreciate your suggestion. We will duly consider this aspect during the comprehensive proofreading process of the entire manuscript.

2. Lines 34-35: Clarify the language here as it is unclear whether you mean data from this particular study or another study.

Pilot study was the preliminary step of the present study. It is neither possible nor relevant to include a more thorough description of it in the Abstract, thus we removed it from this section.

3*. Lines 151-156: It is unclear whether this paragraph is about this study in particular or future directions (not including this study).*

We express our gratitude for your kind attention. The opening sentence of this paragraph will be revised as follows: “The future direction of studies using digital interventions is to specify the extent of which electronic behavioural interventions help reduce pain intensity and psychiatric symptoms.”

4. Move section 2.1 to the introduction.

We appreciate your suggestion. Appropriate changes are made to the manuscript

5. The paragraph starting in 175 seems out of place. I suggest replacing this paragraph with a paragraph highlighting how this study adds to the literature - there have been few (if any) previous studies on comprehensive digital interventions, and it is also important to isolate the effective components of a comprehensive intervention.

Thank you for this comment. Appropriate changes are made to the manuscript.

6. In lines 391-395, the authors mention a small-scale pilot study. It doesn't seem like that study a part of this manuscript (rather, it is a separate study), but the result (that specialist time is reduced more than tenfold) is mentioned in the abstract. If the pilot study was separate from this manuscript, I suggest removing this result from the abstract. If the pilot study was part of this manuscript, I suggest explaining it and the result in much more detail.

We appreciate your comment. The pilot study was performed prior to the present study. The data of the pilot study is unpublished and is a matter of a separate publication. Appropriate changes are made to the manuscript.

Sincerely,
Triinu Niiberg-Pikksööt
Corresponding author
