# Supplementary material for: Implementing a digital solution for patients with migraine—Developing a methodology for comparing digitally delivered treatment with conventional treatment: A study protocol
Source: PLOS Digit Health. 2024 Feb 29;3(2):e0000295. doi: 10.1371/journal.pdig.0000295 (PMC10903846; doi:10.1371/journal.pdig.0000295)
Supplement: Attachment — Submitted filename: Response to Reviewers.pdf [file pdig.0000295.s003.pdf]

Dear Editor and Reviewers

We are sincerely grateful for editor and all the reviewers for their time, comments and expertise on the manuscript "Implementing a digital solution for patients with migraine - developing a methodology for comparing digitally delivered treatment to conventional treatment: A study protocol" by Triinu Niiberg-Pikksööt et al.

Editor points out the following minor issues to revise

1. Please add appropriate keywords using the MeSH terms.

We acknowledge your recommendation and confirm that we revised the existing keywords and added appropriate MeSH terms.

2. Please remove the subheadings from the introduction section and provide an integrated introduction section. Moreover, the introduction section needs to be summarized in 2-3 pages.

We revised the intro to address length issues. The study covers several areas, and in rewriting the introduction, we aimed to maintain scientific rigor while making it more concise. We appreciate your feedback and are thankful for additional input during the review.

3. Overall, the manuscript is very long and I suggest to move tables to the Appendix section, and summerize different sections of the manuscript.

We substantially reduced the volume of the manuscript, but additionally we'd like to clarify the suggestion of the editor moving tables into the Appendix section. However, the Journal requirements state that tables must be inserted immediately after the first paragraph in which they are cited.
If we use appendixes, do the appendixes have to be at the end of the manuscript or should appendixes (each table separately) be presented as a supporting information?

4. The discussion section needs to be supported by more references.

We enhanced the discussion section by incorporating additional references to substantiate our points. We appreciate your guidance in ensuring the quality of our analysis.

Reviewer #1 points out nessessity to write the references uniformly, some have date, other do not have dates.

Thank you for highlighting this discrepancy and we appreciate your diligence in refining the quality of our references. We reviewed references and ensured consistency in referencing format.

We express our gratitude to **Reviewer #2 and Reviewer #3** for their invaluable feedback and prior comments. Following a thorough review, we have reexamined and restructured the discussion section, incorporating your insightful suggestions. Your contributions are greatly appreciated.

Sincerely,
Triinu Niiberg-Pikksööt
Corresponding author
